# In vivo optical imaging-guided targeted sampling for precise diagnosis and molecular pathology

**DOI:** 10.1038/s41598-021-01447-4

**Published:** 2021-11-30

**Authors:** Aditi Sahu, Yuna Oh, Gary Peterson, Miguel Cordova, Cristian Navarrete-Dechent, Melissa Gill, Christi Alessi-Fox, Salvador Gonzalez, William Phillips, Steven Wilson, Reza Afzalneia, Raven Rose, Abu-Akeel Mohsen, Danielle Bello, Ashfaq Marghoob, Anthony Rossi, Jedd D. Wolchok, Taha Merghoub, Veronica Rotemberg, Chih-Shan Jason Chen, Milind Rajadhyaksha

**Affiliations:** 1 Dermatology Service, MSKCC, New York, NY USA; 2 Department of Dermatology, Pontificia Universidad Católica de Chile, Santiago, Chile; 3 Department of Pathology, SUNY Downstate Medical Center, Brooklyn, NY USA; 4 SkinMedical Research and Diagnostics, P.L.L.C., Dobbs Ferry, NY USA; 5 Faculty of Medicine and Health Sciences, University of Alcala de Henares, Madrid, Spain; 6 Caliber Imaging and Diagnostics Inc., Rochester, NY USA; 7 Human Oncology and Pathogenesis Program, MSKCC, New York, NY USA; 8 Ludwig Collaborative and Swim Across America Laboratory, MSKCC, New York, NY USA; 9 Department of Surgery, MSKCC, New York, NY USA; 10 Parker Institute for Cancer Immunotherapy, MSKCC, New York, NY USA; 11 Department of Medicine, MSKCC, New York, NY USA; 12 Weill Cornell Medical College, New York, NY USA

**Keywords:** Molecular medicine, Diagnosis, Medical imaging, Imaging, Biological techniques, Microscopy, Confocal microscopy, Medical research, Translational research, Skin cancer, Cancer imaging

## Abstract

Conventional tissue sampling can lead to misdiagnoses and repeated biopsies. Additionally, tissue processed for histopathology suffers from poor nucleic acid quality and/or quantity for downstream molecular profiling. Targeted micro-sampling of tissue can ensure accurate diagnosis and molecular profiling in the presence of spatial heterogeneity, especially in tumors, and facilitate acquisition of fresh tissue for molecular analysis. In this study, we explored the feasibility of performing 1–2 mm precision biopsies guided by high-resolution reflectance confocal microscopy (RCM) and optical coherence tomography (OCT), and reflective metallic grids for accurate spatial targeting. Accurate sampling was confirmed with either histopathology or molecular profiling through next generation sequencing (NGS) in 9 skin cancers in 7 patients. Imaging-guided 1–2 mm biopsies enabled spatial targeting for in vivo diagnosis, feature correlation and depth assessment, which were confirmed with histopathology. In vivo 1-mm targeted biopsies achieved adequate quantity and high quality of DNA for next-generation sequencing. Subsequent mutational profiling was confirmed on 1 melanoma in situ and 2 invasive melanomas, using a 505-gene mutational panel called Memorial Sloan Kettering-Integrated mutational profiling of actionable cancer targets (MSK-IMPACT). Differential mutational landscapes, in terms of number and types of mutations, were found between invasive and in situ melanomas in a single patient. Our findings demonstrate feasibility of accurate sampling of regions of interest for downstream histopathological diagnoses and molecular pathology in both in vivo and ex vivo settings with broad diagnostic, therapeutic and research potential in cutaneous diseases accessible by RCM-OCT imaging.

## Introduction

Accurate disease diagnosis and molecular profiling in the presence of spatial heterogeneity require high-yield biopsies containing adequate viable pathological tissue with key diagnostic and molecular features. However, the current approach of “visually-guided,” and thus, variable sampling, results in cancer misdiagnoses^1^, necessitating multiple biopsies in about 17–40% of cases^2^. Furthermore, molecular profiling using processed formalin-fixed paraffin-embedded (FFPE) specimens with laser-capture microdissection suffers from inadequate DNA and RNA quality and quantity in ~ 20% and ~ 30% specimens, respectively^3,4^. As morphological and molecular profiling of tumors has the potential to inform diagnoses, prognoses, and drive therapeutic decisions, molecular pathology has become an indispensable tool for patient care in this era of ‘precision medicine’. The current challenges for comprehensive diagnostic and molecular profiling in cancer diagnosis and management are mainly associated with sample quality and/or adequacy, and intratumor heterogeneity^5–7^. Techniques that enable accurate and consistent morphology-guided sampling for diagnosis and molecular assessment are crucial.

Ultrasound, X-ray, computed tomography, magnetic resonance and fluorescence molecular imaging are routinely employed to guide biopsy sites^8,9^; however, the lower spatial and/or absence of temporal resolution impedes visualization of the tissue histomorphology in real-time^10^. Alternatively, high-resolution imaging using reflectance confocal microscopy (RCM) and optical coherence tomography (OCT) can be used to guide sampling. Both RCM and OCT are label-free non-invasive optical imaging approaches that rely on endogenous backscattered contrast. RCM and OCT imaging can detect pathological changes in tissues, and have shown promise for disease diagnosis in dermatology, urology and ophthalmology^11^. Both RCM and OCT can detect and diagnose epithelial and melanocytic neoplasms in skin and the oral cavity with high sensitivity and specificity^12,13^. RCM and OCT provide cellular-level and microstructural-level resolution, and image up to a depth of 0.2 mm and 1 mm, respectively^14,15^, enabling visualization of the underlying cellular morphology in neoplasms and inflammatory disorders at the bedside^12,13^*.* Thus, RCM and OCT can potentially guide detection of the most diagnostic or prognostic areas directly in vivo, allowing for accurate and targeted biopsies for precise downstream histopathology and molecular profiling^10^. Use of OCT imaging-guided needle core biopsies has previously been demonstrated^16^. In this study, we demonstrate novel imaging-guided in vivo spatial targeting and 1–2 mm sampling through reflective metallic grids in skin cancers for diagnosis, feature and tumor-depth correlation, and molecular pathology using Memorial Sloan Kettering-Integrated mutation profiling of actionable cancer targets (MSK-IMPACT)^17^.

## Methods

In vivo patient imaging was conducted on 9 either clinically suspicious or histopathologically confirmed melanocytic (melanoma and lentigo maligna) or keratinocytic tumors [basal cell carcinoma (BCC) and actinic keratosis] in 7 patients. Ex vivo studies were performed on 4 surgically excised BCC specimens. The study was approved by the Institutional Review Board at Memorial Sloan Kettering Cancer Center (MSKCC-IRB). Written informed consent was obtained from all participants. All research was performed strictly in accordance with the Declaration of Helsinki and relevant guidelines and regulations.

### Metallic grid design and manufacturing

We designed an optically reflective metallic grid with square-shaped windows of sizes 0.5, 1.0, and 2.0 mm and wall thickness 50–100 µm, using computer-aided design software (SolidWorks, Dassault Systèmes, France). The metallic grids were custom-manufactured through an electroforming process, using nickel as the base material (Shimifrez, Ontario, Canada). Circular notches etched along the X and Y axes on the grid served as navigational landmarks.

### Grid placement

The metallic grids were centered over the areas of interest and attached using surgical tape (Micropore Surgical Tape [3M, MN, US]) or adhesive Mastisol (Eloquest Healthcare, MI, US). Clinical and/or dermoscopic images were acquired after grid placement.

### In vivo imaging procedure

Imaging was performed using either an RCM (tissue-coupled VivaScope 1500 or handheld VivaScope 3000, Caliber I.D., Rochester, NY) or an integrated handheld RCM-OCT prototype (described elsewhere)^18^. RCM and/or RCM-OCT images were acquired at multiple locations within the lesion for comprehensive lesion evaluation. Between 3–4 mosaics^19^ were acquired at epidermis, dermal–epidermal junction and dermis, followed by stacks and videos focused in the areas of interest. Images were read and interpreted in real-time at the bedside to select the targeted biopsy site(s) by 2 investigators (M.C. and A.S.) with more than 4 years of RCM/OCT reading experience. Images were evaluated for previously described criteria for keratinocytic and melanocytic lesions^20–27^. These features included basaloid nests (hyporeflective ovoid nests) connected to epidermis or in dermis with peripheral palisading and peritumoral mucin, epidermal streaming and stromal changes (for BCC), and keratinocytic atypia with degrees of epidermal disarray with or without vertical and button-hole vessels (for actinic keratosis and squamous cell carcinoma). Pagetoid cells (both dendritic and round) in the suprabasal epidermis, atypical cells at the dermal–epidermal junction, and junctional thickenings with or without presence of intradermal cerebriform nests were evaluated in lesions suspicious for melanoma^20–27^.

### Ex vivo proof-of-concept testing

The 0.5- mm and 1-mm sampling for morphological or molecular analysis were initially tested on 4 ex vivo debulked BCC tissues obtained from Mohs surgery.

### Precision biopsy and histopathology

In the selected grid windows (showing features of interest), biopsies were obtained using sterile micro-sampling punches of 1.0 mm or 2.0 mm diameters (Rapid-Core Sampling Tool [Electron microscopy Sciences, PA] or Integra Miltex [Integra Life Sciences, NJ]). The biopsy specimens were immediately processed for frozen sectioning. Based on frozen histopathology results from the precision biopsy, the remaining lesion was either excised and processed for routine FFPE histopathology or immediately treated. Depending on the clinical question, specimens were embedded either on edge (vertical sections) or *en face* (horizontal sections). Ten-micron thick ribbons of serial sections were prepared and stained with hematoxylin and eosin (H&E).

### Quality control and IMPACT analysis

Samples for DNA extraction and quality control assessments were collected in sterile Eppendorf tubes (Eppendorf, Germany) and immediately transferred to − 20 °C for short-term storage and submitted to Integrated Genomics Operation (IGO) core. DNA from the frozen tissue was isolated with DNeasy Blood & Tissue Kit (QIAGEN catalog #69504) with 1-h of incubation at 55 °C for digestion. DNA was eluted in 0.5X Buffer AE. The quantity and quality of DNA were estimated using Quant-it PicoGreen and Agilent TapeStation D1000. For MSK-IMPACT, 100 ng DNA was used to prepare libraries using KAPA Hyper Prep Kit (Kapa Biosystems KK8504) with 8 PCR-cycles. 100 ng of each barcoded library were captured by hybridization in equimolar pools using the Integrated Mutation Profiling of Actionable Cancer Targets (IMPACT) assay (Nimblegen SeqCap), designed to capture all protein-coding exons and select introns of 505 commonly implicated oncogenes, tumor suppressor genes, and members of pathways deemed actionable by targeted therapies. Captured pools were sequenced on a HiSeq 4000 in a PE100 run using the HiSeq 3000/4000 SBS Kit (Illumina) producing an average of 500X coverage per tumor. The IMPACT data was analyzed on cBioportal^28,29^.

## Results and discussion

### Imaging-guided precision 2-mm biopsy enables accurate targeting and diagnosis in melanocytic and keratinocytic tumors in vivo

**Figure 1:**
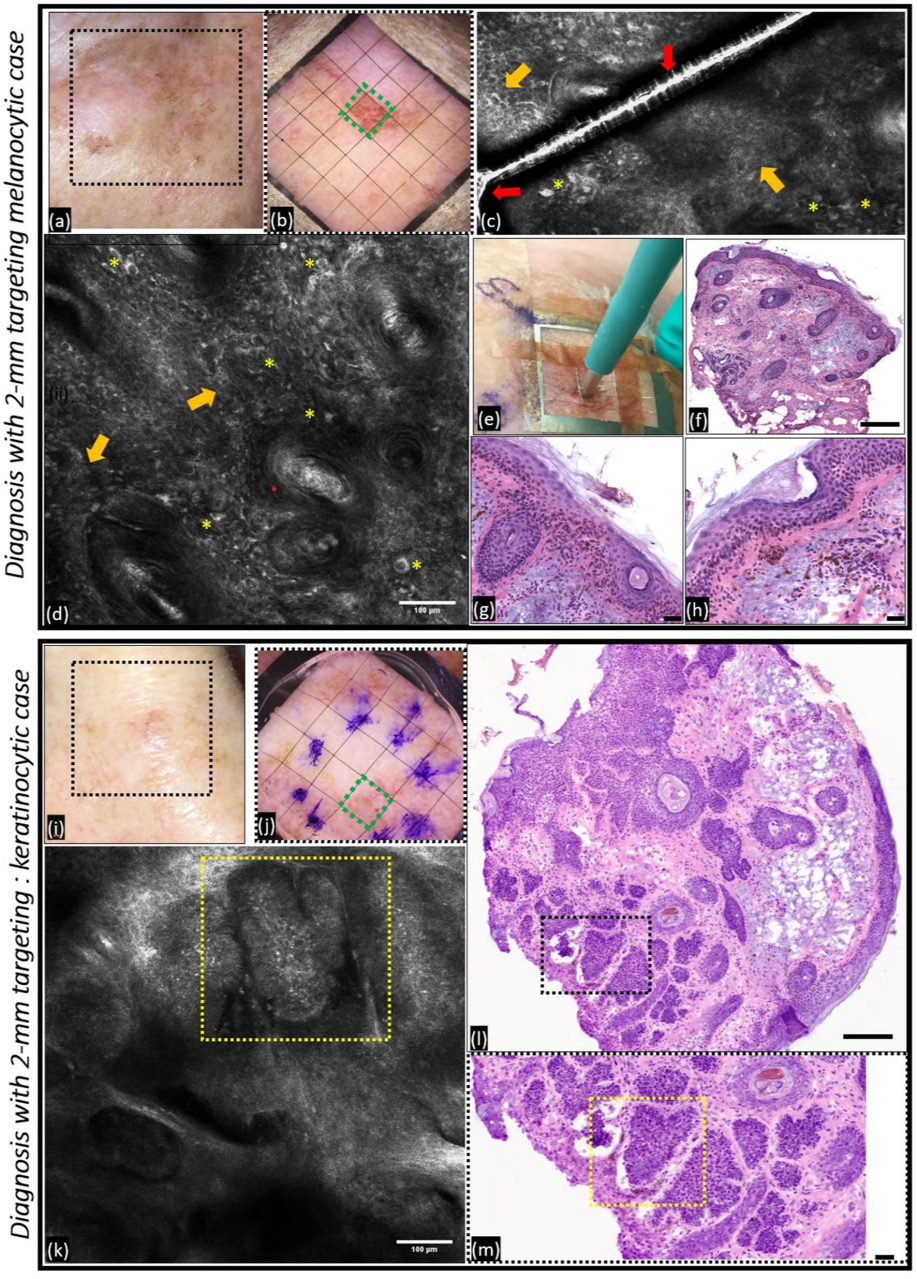
Targeted 2 mm-biopsy enables accurate tumor diagnosis. *Diagnosing a suspected melanocytic lesion.* (**a**) Clinical photograph of an ill-defined pigmented lesion concerning for lentigo maligna (melanoma in situ) on the left cheek and eyelid; (**b**) close-up photograph after placement of the 2-mm grid centered on the lesion (green box indicates the area targeted for biopsy after review of RCM imaging), (**c**) the reflective gridlines (red arrows) in addition to pigmented atypical keratinocytes (yellow asterisks) are visible in the RCM image, (**d**) another RCM image of the epidermis shows pigmented keratinocytes with cytological atypia sparing the adnexal structures (hair follicles) and a notable absence of a melanocytic neoplasm, indicating the presence of pigmented actinic keratosis, a precancerous lesion (instead of the original clinical indication of lentigo maligna); (**e**) a 2-mm biopsy of the area showing the most representative diagnostic features being performed through the window in the grid; (f) vertical frozen histopathology sections showing preserved tissue morphology (10 ×, scale bar = 300 µm); (**g**, **h**) high magnification images showing epidermal hyperpigmentation, partial-thickness keratinocytic atypia and solar elastosis confirming the RCM diagnosis of pigmented actinic keratosis (40 ×, scale bar = 50 µm). The accurate and representative nature of the RCM-guided biopsy was validated by subsequent excisional biopsy, performed as part of the standard of care, which confirmed the diagnosis as pigmented actinic keratosis. *Diagnosing a suspected keratinocytic case.* (**i**) Clinical photograph of a pigmented lesion clinically suspected to be a basal cell carcinoma; (**j**) dermoscopic photograph after placement of the 2-mm grid centered on the lesion (green box indicates the area targeted for biopsy after review of RCM imaging); (**k**) RCM image reveals basaloid tumor islands showing peripheral palisading and a dark rim of mucin (heart-shaped tumor in yellow dotted rectangle); (**l**), 2-mm punch through the grid followed by preparation of horizontal histopathology sections that confirm the presence of BCC (10x, scale bar = 300 µm); (**m**) the inset highlights the heart shaped tumor island seen in RCM images (40 ×, scale bar = 30 µm). This experiment establishes the proof-of-concept for spatial targeting and illustrates accurate diagnostic pathology with high-resolution imaging-guided precision biopsy and minimally invasive sampling.

The proof-of-concept for imaging-guided precision targeting was verified on 2-mm biopsies in 4 patients in vivo*.* Accurate targeting and sampling for diagnosis was investigated in 4 patients with suspected melanocytic (n = 1) and keratinocytic (n = 3) tumors. Within multifocal areas of tumor cells interspersed with normal skin and/or tumor stroma, RCM/OCT imaging identified the “target” area with the most representative diagnostic information in each of these lesions. For example, in the suspected melanoma in situ, lentigo maligna type (Fig. 1a,b), RCM revealed pigmented atypical keratinocytes and the absence of a melanocytic proliferation, suggesting an in vivo diagnosis of pigmented actinic keratosis, a precancerous keratinocytic lesion (Fig. 1c,d). This RCM diagnosis was confirmed on histopathology after precision targeting (Fig. 1e–h) of the atypical keratinocytes. Subsequently, the representative nature of the 2-mm precision biopsy was confirmed by the diagnosis of pigmented actinic keratosis rendered on the biopsy and fixed histopathology performed as the standard of care for atypical pigmented lesion suspicious for melanoma. Although further studies are needed to validate the utility of these precision biopsies, this approach may help avoid traumatic biopsies in such cosmetically or functional sensitive anatomical locations. The 3 keratinocytic tumors were found to be BCC and were promptly treated during the single patient visit. In these cases, precision biopsy and confirmatory frozen section histopathology identified basaloid nests with peripheral palisading of nuclei and peritumoral mucin in the keratinocytic tumors. The representative case (Fig. 1i,j) shows successful targeting of nodular BCC, especially a heart-shaped tumor nest seen in vivo on RCM (Fig. 1k–m).

### Imaging-guided 1-mm precision biopsy enables feature correlation and yields adequate genetic material confirmed on ex vivo surgical specimens

Prior to in vivo testing, the feasibility of 1-mm imaging-guided precision biopsy for feature correlation was verified in ex vivo specimens (Fig. S1). BCC tumor with minute calcifications and subepidermal collection of macrophages were successfully targeted. Subsequently, adequate quantity and quality of DNA were confirmed in 0.5-mm and 1-mm biopsies, towards molecular pathology with high-throughput next-generation sequencing (NGS) in these small precision biopsies (Fig. S2).

### Imaging-guided 1-mm precision biopsy enables diagnosis, micron-size feature correlation and tumor depth evaluation in vivo

**Figure 2:**
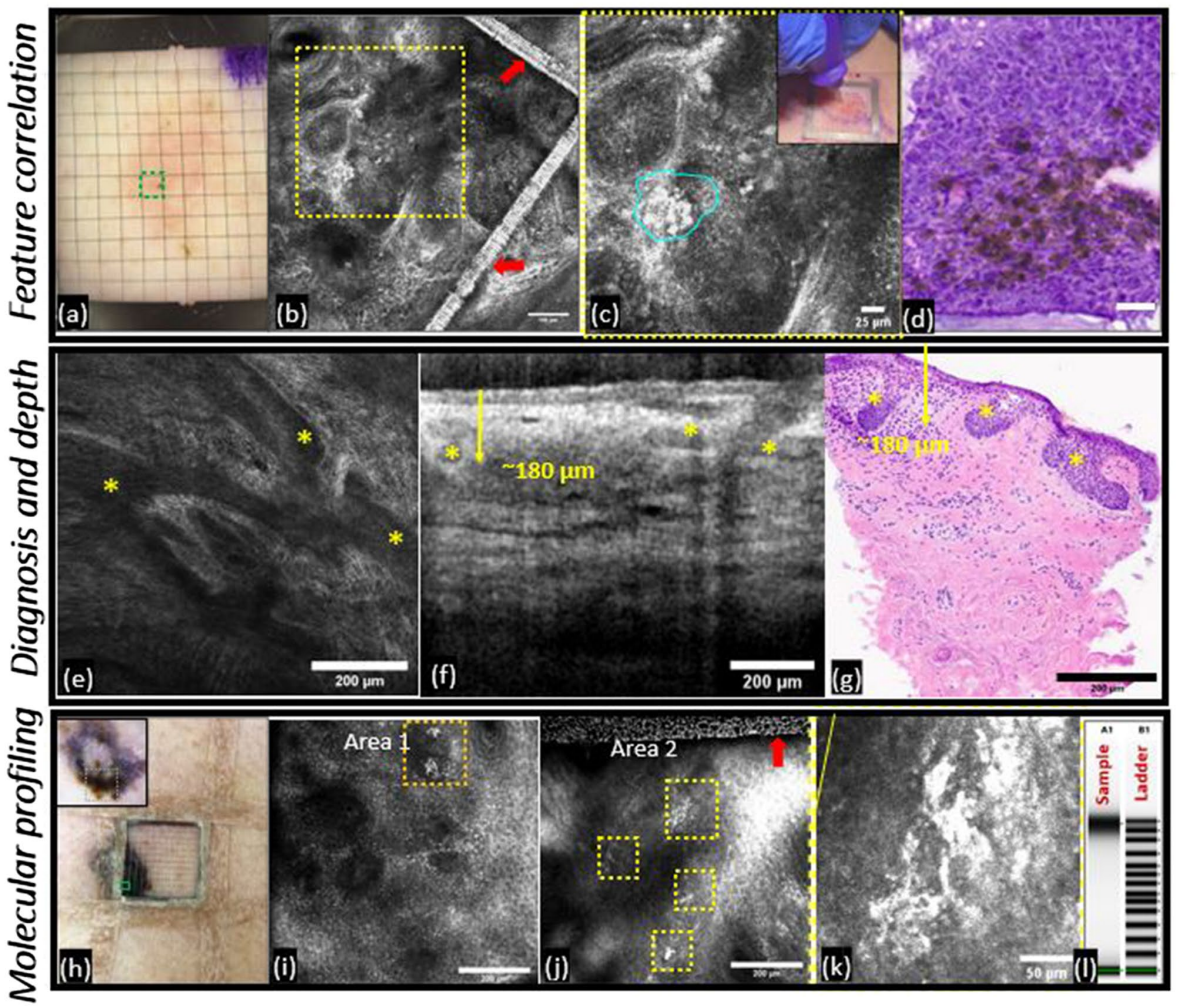
Targeted 1-mm biopsy enables diagnosis, feature and depth correlation, and molecular pathology. *A. Diagnosis and Feature Correlation.* (**a**) Clinical photograph after placing 1-mm grid on a suspected pigmented-BCC (green-box shows targeted-biopsy location following RCM), (**b**) RCM image shows tumor nests with bright cells inside and peripherally, confirming BCC diagnosis (metallic grid lines in RCM shown by red-arrows); (**c**) magnified view of the yellow-square shows bright cells (within cyan-hoops) inside and peripherally to tumor nests with inset photograph demonstrating 1-mm targeted-biopsy for RCM-histopathology correlation; (**d**) frozen histopathology (40x, scale bar = 25 µm) confirms bright cells as melanophages and melanocytes inside and peripheral to BCC nests. *Depth correlation*. (**e**) RCM imaging of suspected keratinocytic tumor revealed nests with peripheral palisading (yellow-asterisk) emanating from epidermis, confirming the diagnosis of BCC; (**f**) the same nests visualized in the superficial dermis on the spatially-registered OCT image with maximum depth measured to be ~ 180 µm; (**g**) precision biopsy followed by vertical sectioning confirmed the RCM-OCT diagnosis and depth. *Molecular Pathology.* (**h**) Clinical photograph of large invasive melanoma with metallic grid (green-box shows targeted-biopsy location following RCM) and dermoscopic photograph as inset (silver area in inset corresponds to grid placement); (**i**) Low density of melanoma cells (orange-boxes) inside tissue volume in area 1, (**j**) high density of melanoma cells (yellow-boxes) in area 2; (**k**) magnified RCM image highlighting the large atypical melanocytes in area 2; (**l**) Targeted 1-mm biopsy from area 2 yielded non-fragmented high-quality DNA (A1, sample) with DNA integrity number (DIN) value of 8.0 and total amount 680 ng which were successfully analyzed with IMPACT505-mutational profiling. These cases illustrate that high-resolution imaging-guided precision biopsy with 1-mm sampling allows correlation of the micron-level features and tumor depth with histopathology and yields adequate quantity and quality of DNA for molecular profiling.

Following the successful ex vivo testing for 1-mm biopsy, in vivo testing for diagnosis, feature and tumor depth correlation was conducted. In the representative case clinically suspicious for BCC (Fig. 2a–d), bright cells within tumor nests in RCM corresponded to the clusters of melanophages and melanocytes within the pigmented BCC on histopathology, demonstrating the feasibility of diagnosis and one-to-one correlation of micron-size imaged features with histopathology. Another tumor clinically suspicious for BCC illustrates the correlation of in vivo imaging features and tumor depth (180 µm) with histopathological diagnosis and depth (Fig. 2e–g).

### Imaging-guided precision 1-mm biopsy facilitates targeted molecular analysis in vivo

Following adequate nucleic acid content in the 1-mm precision biopsies in ex vivo tissues, this approach was tested in vivo in an invasive melanoma. In this patient, RCM enabled identification of low- and high-melanocyte density areas for subsequent targeted biopsy in the high-density area (Fig. 2h–i, Supplementary Video 1). This targeted 1-mm biopsy specimen yielded high DNA quantity (680 ng) and good quality (DIN: 8), optimal for downstream NGS applications, and was successfully analyzed via an MSK-IMPACT505 panel^17^. Missense BRAF V600E oncogenic mutations at 0.02 allelic frequency were found in the IMPACT profiling, qualifying this patient for adjuvant treatments such as RAF inhibitors vemurafenib and dabrafenib, alone, or in combination with the MEK-targeted inhibitors trametinib and cobimetinib, respectively. Thus, this case established the feasibility of in vivo 1-mm precision sampling for downstream next-generation sequencing and facilitated a personalized medicine strategy for improved treatment outcome.

### Imaging-guided 1-mm precision biopsy and mutational profiling indicate differential mutational landscapes across in situ and invasive melanoma

**Figure 3:**
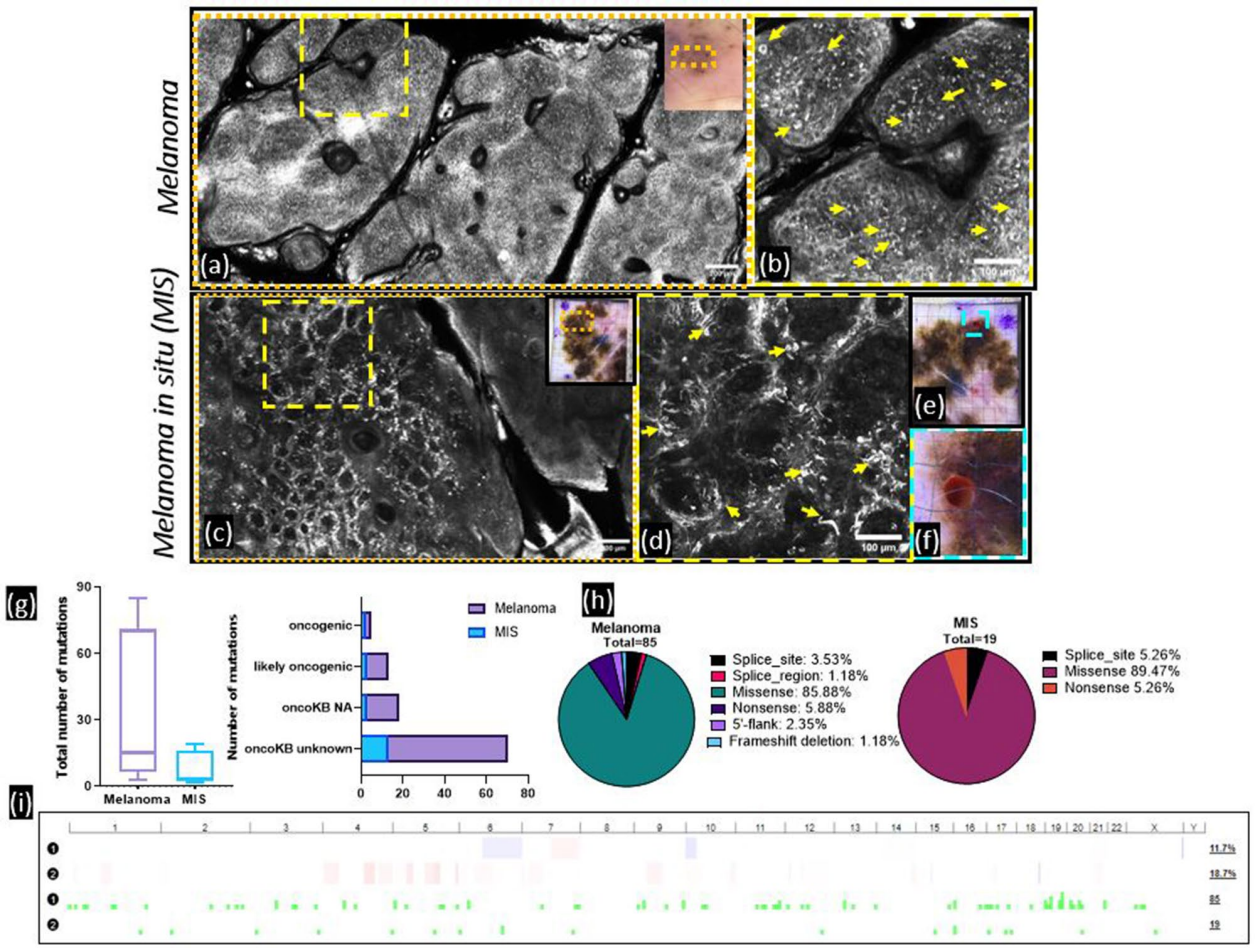
Targeted 1-mm biopsy enables high-throughput mutational profiling that correlates with disease. *Melanoma.* (**a**) RCM imaging within an invasive melanoma (clinical image seen in inset) shows widespread disarray and atypical cells within the entire lesion; (**b**) magnified view of the yellow-square shows large atypical melanocytes in the epidermis (yellow arrows) interspersed with smaller inflammatory cells, including round pagetoid cells that are observed throughout the lesion, along with junctional thickening and melanocyte infiltration in the dermis (not shown in the figure), which are features characteristic of an invasive melanoma. *Melanoma *in situ* (MIS).* (**c**) RCM imaging within a melanoma in situ lesion (clinical image seen in inset) in the same patient shows architectural disorder on the left side as compared to right areas which shows normal skin; (**d**) magnified view of the yellow-square shows clusters of mostly dendritic atypical cells (yellow arrows) at the dermal–epidermal junction, pagetosis in the epidermis (not shown in the figure) with no dermal inflammation, classic features for melanoma in situ; (**e**) dermoscopy image illustrating the 1-mm sampling performed for IMPACT analysis, (**f**) the magnified view shows the 1-mm punch within the grid. *Mutational profiling.* Both melanoma lesions in this single patient were sampled using 1-mm precision biopsies in the most representative areas and subjected to IMPACT analyses on 505 gene panels; (**g**) differential number of total mutations, and variable proportion of oncogenic, likely oncogenic, unknown oncogenic significance and non-oncogenic mutations were found in invasive melanoma and melanoma in situ lesions (total 85 versus 19 mutations, respectively); (**h**) in addition to few splice_site, missense and nonsense mutations, frame-shift deletions, splice_region and 5-flank mutations were also found in the invasive melanoma; (**i**) mutations and copy number alterations mapped over the entire genome in both patients show higher number mutations in melanoma (here, sample 1) but higher fraction of genome altered in the MIS (here, patient 2). This experiment highlights the comprehensive genomic profiling that can be acquired using 1-mm targeted sampling and next-generation high-throughput sequencing.

To confirm cancer stage-specific molecular profiling with 1-mm precision sampling, comparative profiling between a melanoma in situ (MIS) and a primary invasive melanoma (Breslow thickness: 1.5 mm) was performed in a single patient harboring both lesions (MIS on the upper back, invasive melanoma on left upper arm). RCM imaging of the invasive melanoma showcased widespread Pagetoid infiltration by large atypical melanocytes, sheet-like accumulation of atypical melanocytes in the epidermis, junctional thickenings, and large atypical cells in the dermis with cerebriform nests (Fig. 3a,b, Supplementary Video 2), which are typical features for invasive melanoma. RCM imaging identified focal areas with high density of atypical melanocyte clusters at the dermal–epidermal junction, along with Pagetoid cells in the epidermis (Fig. 3c,d, Supplementary Video 3) in the MIS. The most densely populated areas harboring tumor cells were targeted in both lesions; a representative example of the 1-mm biopsy within the grid is shown in Fig. 3e,f. DNA extraction followed by IMPACT profiling for mutations in 505 genes was performed on both lesions. Higher numbers of mutations were observed in the invasive melanoma as compared to the MIS. Similarly, higher frequency of oncogenic and likely-oncogenic mutations was also observed in the invasive melanoma, along with additional mutation types including frameshift deletions (Fig. 3g,h). While the invasive melanoma had a higher number of mutations, a higher fraction of the genome was altered in the MIS (Fig. 3i). Furthermore, 2 statistically significant recurrent hotspot mutations were found in invasive melanoma in CDKN2A and KIT, while in the MIS, only a TP53 hotspot mutation was found. Thus, differential stage-specific mutational landscapes between in situ and invasive melanoma were identified through imaging-guided mutational profiling.

## Discussion and conclusion

Prior studies on clinically (i.e., visually)-targeted biopsies have demonstrated the feasibility of 0.33–0.5 mm biopsies for histology, ultrastructural analysis, and gene expressions evaluation in a small number of genes^30,31^. RCM-guided 2-mm targeted biopsies using *paper-rings* for histopathological correlation have also been demonstrated^32^*.* The findings from our study demonstrate proof-of-concept ability to perform imaging-guided 1-mm targeted biopsies for downstream diagnosis (by preserving tissue architecture) and next-generation molecular profiling (by ensuring high nucleic acid quality and quantity) in both in vivo and ex vivo settings.

Limitations of this approach include the relatively shallow depth of imaging (0.2–1 mm) with limited sensitivity for deeper structures and endogenous backscattered contrast that limits specificity (~ 60–80%) for distinguishing morphologically similar structures. Nevertheless, most cutaneous cancers and inflammatory disorders arise or manifest near the epidermal-dermal junction (0.1–0.2 mm in skin) which can be routinely imaged with both RCM and OCT with high sensitivity(~ 90%)^12,13^. Furthermore, the deeper structures (> 0.2 mm), especially, aberrant vasculature can be visualized by OCT^33^.

The current approach may be improved by developing adhesive reflective grids that obviate the need for external adhesives such as paper tape and surgical adhesives. Furthermore, additional notches may be added to serve as visual cues to improve navigation during in vivo imaging. These visual aids can be introduced in the center and at the outer boundary along the X and Y axis to set up quadrants and a coordinate plane for imaging guidance. Towards these improvements, we are investigating single-use removable custom-made reflective tattoos and the use of 3-D printed “caps,” which can be printed with a central aperture ranging from 0.5 to 2 mm diameter.

Finally, our in vivo imaging approach is immediately implementable for cutaneous cancers and non-neoplastic diseases such as keratinocyte and melanocyte cancers (squamous cell carcinoma, basal cell carcinoma, cutaneous and mucosal melanoma), cutaneous lymphoma, and extra-mammary Paget’s disease, atopic dermatitis, psoriasis, and graft versus host disease. The in vivo approach in skin also may be adaptable for ex vivo applications in diverse tissues, e.g. targeted tissue can be collected for electron microscopy or molecular analysis for precision medicine, from surgical pathology or archived specimens in a biobanking setting^34^. Development of new RCM-OCT device configurations(for example, an intra-oral probe^35^), when integrated with optical tissue-marking approaches (for example, laser ablation or coagulation marking), may allow the in vivo, imaging-guided targeted biopsy approach to be extended into other oncology settings.

In future, this novel imaging-guided technique could potentially transform research and patient care by enabling accurate and adequate sampling for direct correlation between histomorphology and molecular pathology across a lesion at multiple time points, to improve accuracy and consistency of diagnosis and identify therapeutic molecular targets (as shown in Figs. 1, 2). It can also have a role in monitoring cellular-level treatment response by tracking tumor mutation burden (as shown in Fig. 3) or evaluating immune biomarkers such as PD-L1 during treatment. With the help of *small* 1-mm biopsies at multiple locations, multiple time points or longitudinal data can be collected. Additionally, imaging-guided targeting of tumor-enriched areas can allow identification of novel downregulated or upregulated actionable targets that can be missed with conventional bulk-tissue sequencing. In this era of precision medicine, this novel imaging-guided approach may enable broad diagnostic, therapeutic, and research applications in both oncology and inflammatory diseases.

## Supplementary Information

Supplementary Information 1.

Supplementary Video 1.

Supplementary Video 2.

Supplementary Video 3.
