# Supplementary material for: In vivo optical imaging-guided targeted sampling for precise diagnosis and molecular pathology
Source: Sci Rep. 2021 Nov 30;11:23124. doi: 10.1038/s41598-021-01447-4 (PMC8633337; doi:10.1038/s41598-021-01447-4)
Supplement: Supplementary file 1 — Supplementary Information 1. [file 41598_2021_1447_MOESM1_ESM.docx]

***In vivo* optical imaging-guided targeted sampling for precise diagnosis and molecular pathology**

Aditi Sahu^1*^^, Yuna Oh^1^^, Gary Peterson^1^, Miguel Cordova^1^, Cristian Navarrete-Dechent^1,2^, Melissa Gill^3,4,5^, Christi Alessi-Fox^6^, Salvador Gonzalez^5^, William Phillips^1^, Steven Wilson^1^, Reza Afzalneia^1^, Raven Rose^7^, Abu-Akeel Mohsen^8^, Danielle Bello^9^, Ashfaq Marghoob^1^, Anthony Rossi^1^, Jedd D. Wolchok^8,10,11,12^, Taha Merghoub^8,10,11,12^, Veronica Rotemberg^1#^, Chih-Shan Jason Chen^1#^, Milind Rajadhyaksha^1#^

^1^Dermatology Service, MSKCC, New York, NY

^2^Department of Dermatology, Pontificia Universidad Católica de Chile, Santiago; Chile

^3^Department of Pathology, SUNY Downstate Medical Center, Brooklyn, NY

^4^SkinMedical Research and Diagnostics, P.L.L.C., Dobbs Ferry, NY

^5^Faculty of Medicine and Health Sciences, University of Alcala de Henares, Madrid, Spain

^6^Caliber Imaging and Diagnostics Inc., Rochester, NY

^7^Human Oncology and Pathogenesis Program, MSKCC, New York, NY

^8^Ludwig Collaborative and Swim Across America Laboratory, MSKCC, New York, NY

^9^Department of Surgery, MSKCC, New York, NY

^10^Parker Institute for Cancer Immunotherapy, MSKCC, New York, NY

^11^Department of Medicine, MSKCC, New York, NY

^12^Weill Cornell Medical College, New York, NY

^Shared first-authors

#Shared senior-authorship

***Corresponding Author:**

Aditi Sahu, Research Associate,

9207-B Dermatology Service

530E, 74^th^ Street,

David H. Koch Center for Cancer Care at Memorial Sloan Kettering Cancer Center

New York-10021, NY.

**Email:**  [sahua@mskcc.org, aditisahu@gmail.com](mailto:xxxxx@xxxx.xxx) **Phone:** 646-608-1935

**Keywords:** optical imaging, reflectance confocal microscopy; optical coherence tomography; precision biopsy; cancer diagnosis; molecular profiling; IMPACT

**Manuscript word count**: 2243

**References** : 32

**Figures** : 3

**Supplementary material:** 2 figures, 3 videos
